# Reconfirmation of newly discovered risk factors of diabetic peripheral neuropathy in patients with type 2 diabetes: A case-control study

**DOI:** 10.1371/journal.pone.0220175

**Published:** 2019-07-29

**Authors:** Yen-Wei Pai, Ching-Heng Lin, Shih-Yi Lin, I-Te Lee, Ming-Hong Chang

**Affiliations:** 1 Neurological Institute, Taichung Veterans General Hospital, Taichung, Taiwan; 2 Department of Medical Research, Taichung Veterans General Hospital, Taichung, Taiwan; 3 Center for Geriatrics and Gerontology, Taichung Veterans General Hospital, Taichung, Taiwan; 4 Department of Medicine, School of Medicine, National Yang-Ming University, Taipei, Taiwan; 5 Division of Endocrinology and Metabolism, Department of Internal Medicine, Taichung Veterans General Hospital, Taichung, Taiwan; 6 Department of Medicine, School of Medicine, Chung Shan Medical University, Taichung, Taiwan; 7 Department of Neurology, School of Medicine, National Yang-Ming University, Taipei, Taiwan; University of Colorado Denver School of Medicine, UNITED STATES

## Abstract

**Aims:**

The aim of the present study was to investigate the major determinants of diabetic peripheral neuropathy (DPN) in patients with type 2 diabetes (T2D), considering the traditional and newly discovered risk factors, including hypoglycaemia and glycemic variability.

**Methods:**

This retrospective case-control study was conducted in a tertiary care hospital in Taiwan. A total of 2,837 patients with T2D were recruited, medical history and biochemical data were obtained, and patients were screened for DPN using the Michigan Neuropathy Screening Instrument (MNSI). DPN was defined as an MNSI exam score > 2. A stepwise selection of variables was used based on the Akaike Information Criterion (AIC) and the Schwarz Criterion (SC). Multivariate analysis was performed using the identified variables obtained from the stepwise selection.

**Results:**

Among the recruited patients, 604 (21.3%) were found to have DPN. 275 patients with DPN were selected because of longer follow up period before enrollment and complete data of glycemic parameters, and paired with 351 patients with T2D without DPN and matched for age, gender, and diabetes duration. The results of the stepwise selection showed that the presence of moderately and severely increased albuminuria yielded the lowest values of AIC and SC, which indicate the best predictive performance. Multivariate analysis demonstrated that moderately and severely increased albuminuria and greater long-term glycemic variability significantly increased the risk of DPN, with a corresponding odds ratio of 1.85 and 1.61 (95%confidence intervals of 1.25–2.73and1.02–2.55, respectively), after adjusted for hypoglycaemia and types of diabetes treatment.

**Conclusions:**

Albuminuria is a potent predictor of DPN, and greater long-term glycemic variabilityis clearly associated with DPN in adults with T2D. These findings indicate that, in addition to achieve average blood glucose control, screening for albuminuria and reducing blood glucose fluctuations might be useful for improving diabetic microvascular complications.

## Introduction

Diabetic peripheral neuropathy (DPN) is a common and debilitating microvascular complication of diabetes, affects nearly half of patients with diabetes after 10 years, increases the risk of ulcerations and lower extremities amputations, and has a deleterious impact on quality of life [1]. Older age, longer diabetes duration, poor glycemic control, hypertension, and dyslipidemia are well-known risk factors of DPN. Glycated hemoglobin (HbA1c) is considered the gold standard and surrogate end-point for glycemic control in the long-term diabetes management. However, many results from clinical trials and systematic reviews suggest that enhanced sugar control for targeting normal HbA1c levels does not significantly reduce DPN in patients with type 2 diabetes (T2D) but increases the risk of severe hypoglycemic episodes [2]. Given the high prevalence and limited therapeutic options, it is important to identify potentially modifiable risk factors of DPN in individuals with T2D [3]. Hypoglycemia or glycemic variability, an important aspect of overall glycemic control, may provide an additional or better value in predicting diabetic complications than the average glycemic measure. A number of studies suggested that long-term glycemic variability, as determined by visit-to-visit variations in fasting plasma glucose (FPG) or HbA1c, is a potent indicator in patients with T2D [4, 5]. In addition, recent evidence has shown that previous hypoglycemic events requiring hospitalization or visits to emergency department were associated with an increased risk of DPN, independent of HbA1c levels [6]. To date, no study has yet evaluated which of these newly discovered and previously found risk factors play a key role in DPN. Therefore, the aim of this study was to investigate the major determinants of DPN in a large cohort of adult patients with T2D.

## Materials and methods

### Study design and participants

This retrospective case-control study enrolled type 2 diabetes patients aged 20 years and older from the outpatient department of a tertiary care teaching hospital in Taiwan between January and October 2013. The diagnosis of T2D was established according to the criteria of American Diabetes Association (ADA). Patients with type 1 diabetes, gestational diabetes mellitus, follow-up duration <1 year, and less than two records of blood biochemical measurements within six months before enrolment were excluded from this study. Finally, a total of 2,837 patients with T2D were recruited. Prior to analysis, patient data was anonymized by the computer. The study protocol was reviewed and approved by the Institutional Review Board of Taichung Veterans General Hospital (CE-17217B).

### Anthropometric and biochemical measurements

Upon entry into our study, all participants completed a standardized, computerized questionnaire administered by a case management nurse that was used to record their previous or current disease status, medication use, and lifestyle habits and also underwent anthropometric measurements, including height and weight (both recorded using a calibrated balance with a stadiometer). Body mass index (BMI) was calculated by dividing the weight in kilograms by the height in squaremeters (kg/m^2^).

The participants’ laboratory tests and medications were obtained from hospital records and were repeated every three months. After overnight fasting, blood was drawn from an antecubital vein in the morning and sent for collection for biomarkers, such as serum creatinine, fasting plasma glucose ([FPG] using the standard enzymatic method), HbA1c (using high-performance liquid chromatography), and lipid profiles, including triglycerides, high- and low-density lipoprotein cholesterol levels (using the standard enzymatic method). Based on the Modification of Diet in Renal Diseaseequation, the estimated glomerular filtration rate (eGFR) was calculated as 186×(serum creatinine concentration [mg/dL])^−1.154^× (age [year])^−0.203^ (×0.742, if female) [7]. Early morning spot urine samples were obtained to measure urinary albumin and creatinine using the immunoturbidimetric technique and standard enzymatic method (Wako, Osaka, Japan). Urinary albumin-to-creatinine ratio (UACR) was calculated as urinary albumin (mg) divided by urinary creatinine (g). A UACR of≥ 30 mg/g was defined as moderately and severely increased albuminuria [8].

Fasting samples for a glucose assay were obtained every three months during the follow-up period preceding enrollment. The long-term glycemic variability was estimated from the coefficient of variation (CV) of the quarterly visit-to-visit FPG [9, 10]. The study participants were grouped into quartiles according to FPG-CV, and those with a fourth quartile of FPG-CV (≥27.0 in our cohort) were considered to have greater glycemic variations.

### Diagnosis of hypoglycemia and comorbidities

Hypoglycemia was identified bytheInternational Classification of Diseases, Ninth Revision, Clinical Modification (ICD-9-CM) codes 251.0, 251.1, and 251.2 as the main diagnoses during emergency department visits or hospitalization in accordance with the ADA diagnosis. The ADA’s definition of severe hypoglycemia is severe cognitive impairment caused by hypoglycemia, requiring external assistance for recovery without a specific glucose threshold [11]. Hypoglycemic episodes from January 1, 2000toDecember 31, 2012 were included. Patients with ICD-9-CM codes of 270.3 (leucine-induced hypoglycemia), 775.6 (neonatal hypoglycemia), and 775.0 (hypoglycemia in an infant born to a diabetic mother) were excluded from this study.

Any claims, including a history of hypertension (ICD-9-CM codes 401–405), cardiovascular disease (390–438), ischemic heart disease (410–414), chronic kidney disease (582–583) or chronic liver disease (571), for both in and outpatients recorded between2010 and 2012 were considered as comorbidities based on the listed codes.

### Assessment of DPN

DPN was determined using the second component of Michigan Neuropathy Screening Instrument (MNSI), a structured examination of the feet involving an inspection of the feet and evaluation of fine touch (using Semmes-Weinstein 5.07 10-g monofilament), distal vibration perception (using 128-Hz tuning fork), and ankle reflexes. An MNSI exam (MNSIE) score > 2 is diagnostic for DPN. The aforementioned examinations in our study were performed by the same trained and certified investigator in order to control for inter-rater reliability. The investigator only recorded positive (absent response) or negative (normal or diminished response) in items 3–5 (fine touch, vibration perception and ankle reflexes). If more than two positive responses recorded, DPN was determined, with the threshold defined by prior validation studies [12, 13].

### Statistical analysis

Continuous variables were described as the mean±standard deviation (SD), numbers with percentages, or median values with interquartile range. The categorical variables were analysed using Fisher’s exact test or the χ^2^ test, and the continuous variables were analysed using the Student’s t-test or analysis of variance (ANOVA). The CVs of the FPG measurements from outpatient visits before the enrollment of each patient into the study were calculated for those with more than two FPG measurements within six months of enrollment. When considering the effect of the number of visits on CV, the CV of FPG was divided by square root of the ratio of total visits to total visits minus one [4, 14].

A stepwise selection method was performed in order to obtain the best model from a set of predictor variables. Akaike Information Criterion (AIC) and Schwarz Criterion (SC) were used to compare the predictive performance of each model with lower values indicating better predictive performance. The most suitable model was selected as the best model. AIC and SC were both criteria for model selection among a finite set of models, and both of them can be derived from a model’s likelihood function and resulting maximum likelihood estimate. The AIC asymptotically selects the model that minimizes the mean squared error of prediction and also minimizes the maximum possible risk in finite sample size. The SC is consistent in model selection, which could select the true model as the sample size grows as long as the true model is among the candidate models being considered [15]. We used both criteria to test the consistency in these circumstances, whether the true model is in the candidate model set, and also to increase the reliability of our results pertaining to stepwise selection.

A multiple logistic regression analysis was performed using the identified independent variables, and the odds ratios (ORs) between the comparison groups were obtained with 95% confidence intervals (CIs). Statistical significance was defined as a 2-tailed p value <0.05.Statistical analyses were carried out using SAS statistical software version 9.4 for Windows (SAS Institute Inc., Cary, NC).

## Results

### Baseline characteristics

Among the recruited patients, 604 (21.3%) were found to have DPN. 275/604 participants with DPN were further selected because of longer follow up period before enrollment and complete data of FPG and HbA1c testing. 351 non-DPN controls were selected and matched for age, gender and duration of diabetes. Table 1 presents the demographic and clinical characteristics grouped according to the DPN status. The mean age of the study populationwas 72.9 ± 10.5 years, and 53.2% were men. The mean duration of diabetes was 15.4 ± 8.6 years. The majority of participants (97.7%) were treated with pharmacological therapy for diabetes, and 28.4% were treated with insulin (alone or in combination with oral antidiabetic drug). The biochemical parameters were mean values over the follow-up period before enrollment, and the mean FPG and HbA1c level were141.2 ± 43.4 mg/dL and 7.4 ± 1.4% (57 ± 15.3mmol/mol), respectively. The values for each quartile of FPG-CV were ≤12.3, 12.3–18.2, 18.2–27.0, and>27.0. On the basis of the univariate analysis, insulin use (*P* = 0.001), prior severe hypoglycemic episode(s) (*P* = 0.008), greater FPG-CV (*P*<0.001), higher creatinine levels (*P* = 0.001), lower GFR (*P* = 0.001), the presence of albuminuria (*P*<0.001), and larger amounts of albuminuria (*P* = 0.001) were significantly associated with DPN. There were no differences in smoking, alcohol consumption, height, weight, BMI, mean FPG, HbA1c levels or lipid profiles between patients with and without DPN.

**Table 1:** Clinical characteristics of the study participants, according to DPN status, matched for age, gender and diabetes duration.

Variables	Total (n = 626)	Without DPN (n = 351)	With DPN (n = 275)	*P*
Age, years, mean (SD)	72.9 (10.5)	72.5 (10.4)	73.5 (10.7)	0.243
Male, n (%)	333(53.2)	186(53.0)	147 (53.5)	0.908
Smoking, n (%)	28(4.5)	17(4.8)	11 (4.0)	0.612
Alcohol drinking, n (%)	14(2.2)	8(2.3)	6(2.2)	0.566
Height, cm, mean (SD)	161.0 (8.3)	160.8 (8.2)	161.3 (8.5)	0.424
Weight, kg, mean (SD)	65.4 (12.5)	65.2 (11.5)	65.6 (13.7)	0.683
Body mass index, kg/m^2^, mean (SD)	25.1 (3.9)	25.2 (3.7)	24.8 (3.9)	0.935
Diabetes duration, years, mean (SD)	15.4 (8.6)	15.1 (8.1)	15.9 (9.1)	0.294
Diabetes treatment, n (%)				
No medication	13(2.3)	6(1.9)	7 (2.8)	0.001
OAD only	393(69.3)	244(76.3)	149 (60.3)	
Insulin only	56(9.9)	26(8.1)	30 (12.1)	
Insulin + OAD	105(18.50)	44(13.8)	61 (24.7)	
Duration of insulin use, years, mean (SD)	5.5 (4.7)	5.6 (5.3)	5.5 (4.3)	0.925
Hypertension, n (%)	530(84.7)	291(82.9)	239 (86.9)	0.168
Cardiovascular disease, n (%)	568(90.7)	314(89.5)	254 (92.4)	0.214
Ischemic heart disease, n (%)	266(42.5)	141(40.2)	125 (45.5)	0.184
Chronic kidney disease, n (%)	58(9.3)	27(7.7)	31 (11.3)	0.125
Chronic liver disease, n (%)	238(38.0)	144(41.0)	94 (34.2)	0.080
Hypoglycemia, n (%)	35(5.6)	12(3.4)	23 (8.4)	0.008
1	25 (4.0)	9 (2.6)	16 (5.8)	0.027
≥ 2	10 (1.6)	3 (0.9)	7 (2.5)	
Hypertensive drug treatment, n (%)	516(82.4)	282(80.3)	234 (85.1)	0.121
Lipid-lowering agent treatment, n (%)	391 (62.5)	228(65.0)	163 (59.3)	0.145
FPG, mg/dL	141.2 (43.4)	142.7 (41.7)	139.3 (45.4)	0.347
HbA1C, % (mmol/mol)	7.4±1.4 (57±15.3)	7.4±1.3 (57±14.2)	7.5±1.4 (58±15.3)	0.592
Number of FPG testing, n, mean (SD)	20.7 (15.1)	20.2 (14.9)	21.3 (15.2)	0.364
Number of HbA1c testing, n, mean (SD)	32.3 (11)	32.9 (10.5)	31.6 (11.5)	0.119
Follow up duration of FPG testing, years, mean (SD)	4.0 (1.8)	4.1 (1.8)	3.9 (1.8)	0.151
Follow up duration of HbA1c testing, years, mean (SD)	8.0 (2.1)	8.2 (2.0)	7.7 (2.1)	0.002
FPG-CV, %, mean (SD)	20.3 (10.5)	18.3 (9.7)	22.8 (11.0)	< 0.001
≤ 12.3	158 (25.2)	112 (31.9)	46 (16.7)	< 0.001^a^
12.3 to 18.2	154 (24.6)	93 (26.5)	61 (22.2)	
18.2 to 27.0	159 (25.4)	84 (23.9)	75 (27.3)	
> 27.0, n (%)	155 (24.8)	62 (17.7)	93 (33.8)	
Creatinine, mg/dL, mean (SD)	1.3 (1.0)	1.2 (0.8)	1.5 (1.2)	0.001
GFR, ml/min/1.73 m^2^, mean (SD)	64.8 (28.3)	68.6 (26.9)	60.2 (29.3)	0.001
UACR, mg/g, mean (SD)	297.8 (841.2)	186.5 (654)	436.2 (1012.1)	0.001
≥ 30, n (%)	251 (49.5)	115 (40.9)	136 (60.2)	< 0.001
LDL-C, mg/dL	97.8 (27.9)	98.4 (27.7)	97.0 (28.2)	0.558
HDL-C, mg/dL	50.8 (14.6)	50.8 (14.7)	50.9 (14.5)	0.937
TG, mg/dL	137.6 (92.5)	138.3 (85.8)	136.7 (100.2)	0.846

Data are expressed as mean ±SD for continuous variables and frequency (%) for categorical variables.

Difference in continuous variables was analyzed using the Student t test or ANOVA test. Difference in categorical variables was examined by the Fisher’s Exact test or χ^2^ test.

CV: coefficient of variation; DPN, diabetic peripheral neuropathy; FPG: fasting plasma glucose; GFR: glomerular filtration rate; HbA1c: glycated hemoglobin; HDL-C: high-density lipoproteincholesterol; LDL-C: low-density lipoproteincholesterol; OAD: oral antidiabetic drug; SD: standard deviation; TG: triglyceride; UACR: urine albumin-creatinine ratio.

^a^ The *P* value is for fourth quartile of FPG-CV.

### Results of stepwise selection of variables

Table 2 shows the results of the stepwise selection of variables based on AIC and SC. A higher level of HbA1c (defined as HbA1c ≥ 7%) was used as baseline, and variables with lower AICs and SCs were selected. The selected variables were moderately and severely increased albuminuria (AIC 682.186, SC 690.643), type of diabetes treatment (AIC 767.708, SC 785.069), greater FPG-CV (AIC 841.057, SC 849.936), and prior severe hypoglycemic event(s) (AIC 855.450, SC 864.329). The presence of moderately and severely increased albuminuria showed the lowest AIC and SC scores, which indicates the best performance of risk prediction for a given outcome.

**Table 2:** Results of stepwise selection of variables based on AIC and SC.

Model-X variables	AIC	SC
UACR (mg/g) (< 30/ ≥ 30)	682.186	690.643
Type of diabetes treatment	767.708	785.069
FPG-CV (≤ 27.0/ > 27.0)	841.057	849.936
Hypoglycemia (No/Yes)	855.450	864.329
HbA1c (%) (< 7/ ≥ 7)	862.568	871.446

AIC: Akaike Information Criterion; CV: coefficient of variation; FPG: fasting plasma glucose; HbA1c: glycated hemoglobin; SC: Schwarz Criterion; UACR: urinary albumin-to-creatinine ratio.

### Adjusted ORs of DPN using the identified variables

Based on the results of stepwise selection, Table 3 shows the multivariate adjusted ORs and 95% CIs of DPN in patients with T2D. After controlling the competing risk factors, multivariate logistic regression analysis showed that the presence of prior severe hypoglycemic events (OR 2.32, 95% CI 0.96–5.59), moderately and severely increased albuminuria (OR 1.98, 95% CI 1.38–2.85), and greater glycemic variability (OR 1.78, 95% CI 1.02–2.55) were associated with increased DPN risk. After further adjustment for type of diabetes treatment, the presence of moderately and severely increased albuminuria and greater glycemic variability remained significantly correlated with DPN with a corresponding ORs of 1.85 and 1.61 (95% CI 1.25–2.73and 1.02–2.55,respectively).

**Table 3:** Adjusted odds ratio for the association of DPN with the variables according to the result of stepwise selection.

Variable	Adjusted OR ^a^	95% CI	*P*	Adjusted OR ^b^	95% CI	*P*
UACR						
< 30	1.00	─	─	1.00	─	─
≥ 30	1.98	(1.38–2.85)	< 0.001	1.85	(1.25–2.73)	0.002
FPG-CV						
≤ 27.0	1.00	─	─	1.00	─	─
> 27.0	1.78	(1.18–2.70)	0.006	1.61	(1.02–2.55)	0.043
Hypoglycemia						
No	1.00	─	─	1.00	─	─
Yes	2.32	(0.96–5.59)	0.060	1.64	(0.65–4.17)	0.295

CI: confidence interval; CV: coefficient of variation; DPN: diabetic peripheral neuropathy; FPG: fasting plasma glucose; OR: odds ratio; UACR: urinary albumin-to-creatinine ratio.

^a^Model I: Adjusted for UACR (< 30, ≥ 30), FPG-CV (≤ 27.0, > 27.0), hypoglycemia (No, Yes).

^b^Model II: Based on Model I, additionally adjusted for type of diabetes treatment.

## Discussion

This is the first large-scale study to report the major determinants associated with the risk of DPN in adult patients with T2D, considering the newly discovered risk factors, including both hypoglycemia and glycemic variability. The present study demonstrated that the presence of moderately and severely increased albuminuria is an important predictor of DPN, and greater glycemic variability as defined by FPG-CV is a risk factor for DPN in adults with T2D.

It is widely accepted that older age, longer diabetes duration, and poor glycemic control are important risk factors in DPN development [5]. However, multiple clinical trials have revealed that strict blood sugar management, using the average glycemic measure (HbA1c) as therapeutic target, does not stop onset and progression of DPN in patients with T2D [2]. This suggests that additional factors other than mean HbA1c values may account for increased neuropathy risk. Besides, hyperglycemia, hypoglycaemia, and glycemic variations also contribute to the net balance of glycemic status and have emerged as measure of glycemic control. Recent results have suggested that hypoglycemia or glycemic variability may be an additional or better glycemic parameter than mean HbA1c levels for assessing the risk of microvascular and macrovascular complications in patients with diabetes, including DPN [6, 14, 16].

In order to identify the most potent modifiable risk factor for DPN, we conducted a case-control study (matched for non-modifiable risk factors, includingage, gender, and diabetes duration), and we took into account the recently found risk factors. Based on the univariate analysis, only the presence of prior severe hypoglycemic episode, larger amounts of albuminuria, higher level of creatinine, lowereGFR, greater FPG-CV, and modalities for treatment were significantly associated with DPN. The selected variables based on stepwise selection were moderately and severely increased albuminuria, greater FPG-CV, prior severe hypoglycaemic episode(s), and types of diabetes treatment. After controlling the competitive factors in the multivariate analysis, moderately and severely increased albuminuria, and greater FPG-CV remained independently correlating with DPN. The presence of prior severe hypoglycemic episodes showed a trend toward increased risk of DPN but were not significant. The possible explanation indicates that hypoglycemia is one of the most common adverse reactions of diabetes therapy. Excessive lowering of blood glucose level during diabetes therapy, which frequently involves augmenting insulin effects directly or indirectly, might cause hypoglycemia and sometimes severe hypoglycemia. Therefore, there are strong correlations between hypoglycemia and modalities for treatment, and the significance might considerably attenuate if adjusted for both variables.

Our findings are crucial for the clinical management of diabetes. Screening diabetic patients for albuminuria may help identify individuals who are at increased risk of DPN. Besides, controlling only HbA1c without controlling glucose variations may not be sufficient. Avoidance of hypoglycemic episodes over time should be a goal in preventing development or halting of DPN progression.

DPN pathogenesis is complex and multi-factorial, involving complicated mechanisms, including excess glycolysis, augmented flux through the polyol pathway, enhanced activity of hexosamine pathway, overproduction of precursors of advanced glycation end products, and impaired insulin signaling that lead to oxidative stress generation and inflammatory injury [1]. Albuminuria, the pathological excretion of urinary albumin, reflects a state of vascular inflammation and generalized endothelial dysfunction causing further damage to microvessels. Several studies have demonstrated that albuminuria is not only an early marker of renal injury but also be a useful target for therapy because it is potentially reversible. The more albuminuria is lowered, the more beneficial renal and cardiovascular outcome is obtained [17, 18].

Increasing evidence shows that many of the cellular processes that occur with hyperglycemic spikes also occur in hypoglycemic troughs [19]. Several animal studies have reported that experimental hypoglycemia induced by injections of excess insulin in rats resulted in Wallerian-type axonal degeneration [20]. The presently available data suggest that hypoglycemia, rather than hyperinsulinemia, is responsible for insulin-induced hypoglycemic peripheral neuropathy. Evidence suggests that insulin is beneficial for peripheral nervous system (PNS) function and serves a promoter of axonal regeneration after injury, whereas hypoglycemia during normal insulin levels causes axonal degeneration in the PNS. Depletion of energy within neurons due to hypoglycemia may result in both altered intra-neural concentrations of various metabolites, leading to axonal degeneration and myelin breakdown in Schwann cells. Endoneurial microvascular changes caused by local ischemia may also play a role in PNS damage [21]. In addition, oscillating glucose has been shown to be more detrimental to endothelial function than inflammatory cytokines and oxidative stress than chronic sustained hyperglycemic levels, which are involved in the pathogenesis of microvascular complications [22].

The strengths of our study include the large sample size, standardized data collection procedures, detailed information available on possible confounding factors, laboratory test values, and diabetes treatment. Another strength is the use of the validated clinical screening instrument (MNSI), which is sensitive, specific, non-invasive, and easy to administer. There were limitations. First, causality cannot be inferred due to cross-sectional design. Second, the DPN diagnosis was not confirmed byelectrophysiological tests, such as nerve conduction studies. Third, the present study included only Chinese population with T2D, so the result might be not representative of all patients with diabetes mellitus. Fourth, the influence of minor hypoglycemic episodes on the risk of DPN was not assessed in this study.

## Conclusions

In conclusion, our study shows that the presence of moderately and severely increased albuminuria, defined by a UACR of ≥30mg/g, is a strong predictor of DPN. Greater long-term glycemic variability determined by FPG-CV is clearly associated with DPN in adults with T2D. Longitudinal studies are required to confirm the merit of albuminuria or glycemic variability for identifying individuals at increased risk for DPN within the large population of patients with T2D.

## Supporting information

S1 Appendix Part of our dataset. Some information of the participants was not provided due to patient privacy.
(XLS)
